# Research on a Corrosion Detection Method for Oil Tank Bottoms Based on Acoustic Emission Technology

**DOI:** 10.3390/s24103053

**Published:** 2024-05-11

**Authors:** Weixing Hua, Yan Chen, Xiang Zhao, Jiping Yang, Han Chen, Zhaojie Wu, Gang Fang

**Affiliations:** 1 Department Petr Oil & Lubricants, Army Logistics Academy, Chongqing 401331, China; huaweixing@163.com (W.H.); lqylx351@163.com (X.Z.); lqylx561@163.com (H.C.); georgewu2019@163.com (Z.W.); 18986966529@163.com (G.F.); 2 Chongqing Ceprei Industrial Technology Research Institute Co., Ltd., Chongqing 401332, China; lqylx451@163.com

**Keywords:** acoustic emission (AE), oil storage tank, corrosion, detection

## Abstract

This paper presents an acoustic emission (AE) detection method for refined oil storage tanks which is aimed towards specialized places such as oil storage tanks with high explosion-proof requirements, such as cave oil tanks and buried oil tanks. The method utilizes an explosion-proof acoustic emission instrument to detect the floor of a refined oil storage tank. By calculating the time difference between the defective acoustic signal and the speed of acoustic wave transmission, a mathematical model is constructed to analyze the detected signals. An independent channel AE detection system is designed, which can store the collected data in a piece of independent explosion-proof equipment, and can analyze and process the data in a safe area after the detection, solving the problems of a short signal acquisition distance and the weak safety protection applied to traditional AE instruments. A location analysis of the AE sources is conducted on the bottom plate of the tank, evaluating its corrosion condition accurately. The consistency between the evaluation and subsequent open-tank tests confirms that using AE technology effectively captures corrosion signals from oil storage tanks’ bottoms. The feasibility of carrying out online inspection under the condition of oil storage in vertical steel oil tanks was verified through a comparison with open inspections, which provided a guide for determining the inspection target and opening order of large-scale oil tanks.

## 1. Introduction

Oil storage tanks are one of the most crucial pieces of petrochemical equipment and are widely used worldwide, and the most common oil storage tanks are metal ones. However, metal oil storage tanks are characterized by large volume, centralized placement, inflammability, explosiveness, and detection difficulties. There is no doubt that fire and explosions caused by oil leakage will result in huge losses of life and property, as well as serious amounts of environmental pollution [1,2,3]. Storage tank corrosion primarily includes external corrosion and internal corrosion: the former arises from the aging of surface anti-corrosion layers and the corrosive interaction between the tank floor and base layer; the latter encompasses oil- and gas-induced corrosion on top of the tank, corrosion within the gas–liquid fluctuation area, and floor-related corrosive processes. Detecting external corrosion is relatively straightforward with elimination measures available accordingly, while directly identifying internal corrosion, particularly harmful bottom-level corrosion in oil storage tanks, faces tough challenges. Routine inspection by opening tanks is considered to be a direct and effective approach, but it also shows a certain blindness as approximately half of opened oil storage tanks are found to lack obvious defects. Furthermore, it brings about shutdowns, emptying procedures, replacements and cleanings, resulting in substantial economic losses and potential environmental pollution.

AE technology has been used to detect defects in oil storage tanks for many years, and has achieved rich results [4,5], but there are still the following problems: First, there is a lack of explosion-proof detection equipment, which brings a certain explosion risk, and according to the publicly available information, there is no specific solution. Second, due to the complexity and time-consuming nature of open-can detection, the possibility of performing comparisons between the results of the oil tank AE detection and the open-can detection is lower, and a comparative analysis of the verified results is lacking. Third, the results of the oil tank AE detection results needs to be further verified. Compared with ordinary storage tanks, AE instruments designed for use in refined oil storage tanks must meet extremely strict requirements in terms of their explosion-proof performance. Only a few papers have proposed safety levels and evaluation methods for the acoustic emission detection of oil tanks’ defects, but there are few studies on how to carry out safety detection in oil tanks [6,7,8]. The working principle of an explosion-proof AE instrument is introduced in this paper. Using this AE instrument, ten oil storage tanks are tested, and four of them are opened and re-checked. The data were analyzed to derive the location of the acoustic signals for the tank floor corrosion and to assess the integrity of the tank floor.

## 2. Mathematical Model for AE Detection in Tanks

### 2.1. Principle of AE Detection

**Figure 1:**
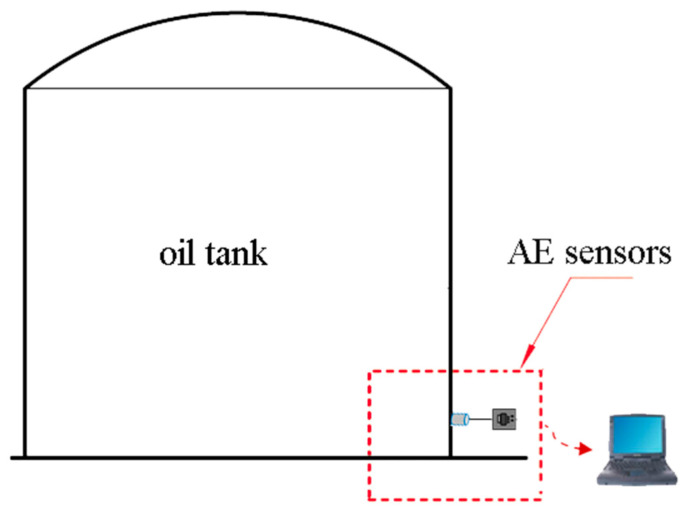
AE principle of an oil tank.

AE refers to elastic strain waves generated by a sudden release of strain energy inside an object [9,10,11,12]. A slight deformation occurs in a corroded area on the bottom plate of a metal storage tank under load pressure, leading to cracking in the corroded layers and the generation of AE signals. Additionally, eddy currents due to leakage from the oil tank’s bottom plate also produce AEs. When using AE technology for detecting defects oil tanks, a predetermined number of piezoelectric ceramic sensors are placed at a uniform angle and circumferential direction around a predetermined height (typically 20–40 cm) above the tank’s bottom wall surface to capture signals originating from its bottom plate, as schematically shown in Figure 1. To identify both quantity and location information about these sources on the tank’s bottom plate, sensors receive signals and transfer them to dedicated equipment responsible for acquiring such signals.

### 2.2. Mathematical Modeling of AE Detection

**Figure 2:**
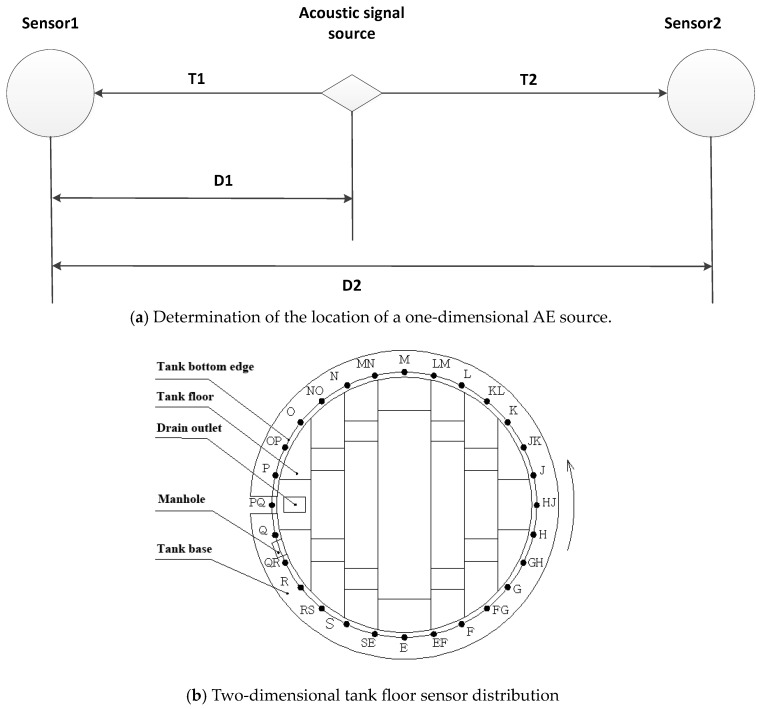
AE detection of tank bottom plate. (E/F/G/…/S represents forward arrangement of sensor location, EF/FG/GH/…/SE represents reverse arrangement of sensor location.).

AE technology is widely used in engineering fields such as storage tanks, pipelines, rock formations and bridges, and its development cannot be separated from the construction of theoretical models [13,14,15,16,17]. The bottom of an oil tank can be regarded as a two-dimensional circular plate, as shown in Figure 2. When the stress on the bottom of the oil tank changes, the measured defect site will generate continuous or discontinuous AE signals. Information about the location of AE signals can be acquired by obtaining the distance and time taken for the signals to reach three or more AE sensors [18,19,20,21,22]. Assuming the signal transmitted from the defect position reaches sensor i after $t_{i}$, and the transmission speed of sound in the tank bottom plate is v, the following formula can be obtained:

(1) $$L_{i}=vt_{i}$$

where $L_{i}$ is the distance between the sensor i and the signal source. When the transmission speed v and sound difference ∆t are known, the distance between the sensor’s position and the AE signal source can be obtained. If the AE signal received by the sensor is $D_{i}$, the corresponding mathematical formula is as follows:

(2) $${D_{i}=ks(t)+v∆t}_{ij}$$

where s(t) is the AE signal source, k is the attenuation coefficient and ${∆t}_{ij}$ is the time difference between the different sensors. Within the corresponding T, the relationship between the signal source information obtained by multiple sensors is as follows:

(3) $$R_{ij}\left({∆t}_{ij}\right)=\frac{1}{T}\int_{0}^{T}ks(t)(t-{∆t}_{ij})dt$$

where $R_{ij}\left({∆t}_{ij}\right)$ is the signal propagation time difference, the position where two AE signals are most correlated.

In a one-dimensional model, the propagation time difference between two AE signals is required, and the location of the AE signal sources can be located in combination with the propagation speed. The relationship between different signal sources can be obtained through the delay time, so the peak point of the signal represents the maximum correlation position between different signal sources, and the information about the position of the defect on the plane of the fuel tank can be calculated using the signals of at least three groups of AE sources.

The sensor layout is based on the maximum detection distance of the sensor, and the maximum sensor spacing in this test is 12 m. In this test, 6–9 m is generally selected and the exact spacing is determined according to the circumference of the oil tank. The one-dimensional model is shown in Figure 2a. The arrangement of AE detection sensors on the tank floor is actually divided into two groups: forward arrangement and reverse arrangement. The two-dimensional tank floor sensor distribution is shown in Figure 2b.

## 3. Design of AE Detection for Tank Bottoms

### 3.1. Tank Inspection Procedure

In AE applications, inspection preparation is critical, especially for the inspection of specialized equipment [23,24,25,26]. The procedures for utilizing an AE instrument to detect oil storage tanks are as follows:

(1) Pressurize the fluid in the oil tank to a static pressure ranging from 60% to 80% below the safe liquid level.
(2) Turn off external tank systems and accessories for at least 12–24 h.
(3) Arrange piezoelectric ceramic sensors at a uniform angle along the outer wall of the oil tank at a distance of about 0.2 m above the bottom plate.
(4) Conduct a pressure-holding test on the oil tank which lasts for between one and four hours.
(5) Perform data processing and analysis on signals captured by the AE instruments to determine both the number of AE sources and the position of the oil tank’s bottom plate. The safety assessment and overall judgment of the tank are based on its corrosion classification standard.

### 3.2. Evaluation of Oil Tank Corrosion Classification

Time difference positioning analysis is conducted on AE sources originating from different areas of interest within a square or circular evaluation area, which should not exceed more than 10% of its diameter. The number of positioning events (E) per hour is calculated after conducting local amplification analysis on all concentrated positioning groups within this evaluation area.

According to the previous AE test results for a large number of oil tanks with the same specifications, the standard AE value (C) value of this test was determined to be five. The C value in Table 1 was determined by using the same testing instrument, setting the working parameters and conducting a certain number of test experiments and opening verification experiments on storage tanks with the same specifications and operating conditions. Based on the time difference positioning results and the criterion AE value (C), each evaluation area’s effective AE source level can be classified accordingly [27,28].

## 4. AE Test Experiments on Tank Bottoms

### 4.1. The Components of the AE Instrument

**Figure 3:**
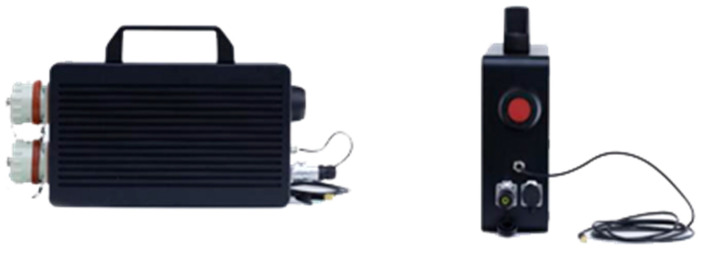
Explosion-proof AE instrument.

The explosion-proof AE instrument used in this experiment is shown in Figure 3. The instrument incorporates an independent signal acquisition channel, a signal synchronization channel, a high-speed acquisition card, a lithium battery pack, a signal amplifier, a data processing system and a cache system. Its shell was constructed using an explosion-proof metal material and it was equipped with developed signal processing software.

**Figure 4:**
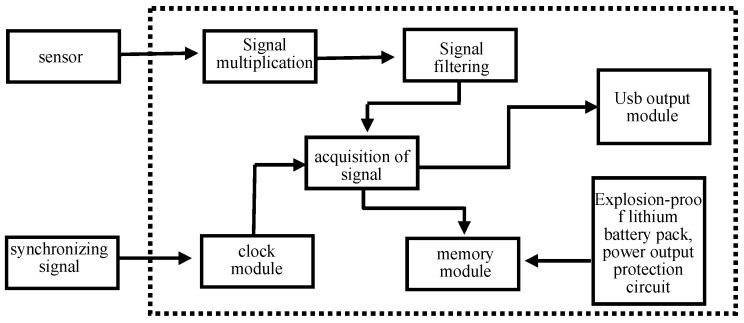
Logic structure of explosion-proof AE instrument.

A Each AE instrument is equipped with a megahertz crystal oscillator to provide a high-frequency synchronous clock signal. In the detection process, one of the AE instruments is designated as the main piece of equipment, and the synchronous cable connected to the main piece of equipment controls the synchronous sampling clock of the other AE instruments to ensure the synchronization of each AE instrument when collecting data. In the process of signal acquisition carried out by the system, the AE signal collected by the sensor is amplified by the signal amplification module inside the instrument, and it is then filtered by the signal filtering module to filter the waveform of the corresponding frequency, and finally, the high-speed acquisition module processes the signal data and stores them on the compact flash (CF) card. The logic structure of the AE instrument is shown in Figure 4.

### 4.2. Performance Parameters of the AE Instrument

**Figure 5:**
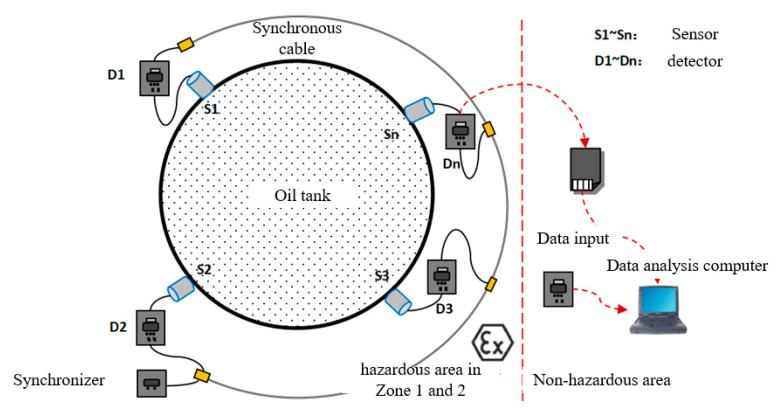
An independent channel AE detection system.

When conducting AE detection for oil tank bottom plates in the level-one explosion-hazard area of oil storage tank cave depots, it is necessary for the internal electrical components of the AE instrument to meet certain explosion-proof standards. Additionally, the AE detection data must be sent to a safe location (60–120 m away from the oil tanks). Due to energy loss during cable transmission, it is necessary for traditional multi-channel AE instruments to utilize 9–12 long-distance cables to simultaneously transmit sensor-collected data, resulting in a higher amount of system power consumption and weaker acquisition signals. To address this issue, an independent channel AE detection system was used for this experiment, as shown in Figure 5.

The system employs multiple AE instruments that are strategically positioned around the oil tank. In addition, a synchronous cable connection synchronizer ensures the synchronization of signals collected by all of the AE instruments. Each AE instrument is equipped with a group of explosion-proof lithium batteries and a data storage module. The AE data can be initially collected and then output to a safe area for postprocessing. Alternatively, the data can be transmitted to signal processing software on a computer in a safe area via long-distance data transmission cables for analysis. The AE instrument detection system used in this experiment effectively addresses the issues associated with a long transmission distance and poor signal acquisition encountered with traditional systems, while taking the safety concerns in an explosion-proof oil tank environment into account.

To enhance the explosion-proof performance of the AE instrument, components such as an FRT-FB183S01C explosion-proof lithium battery pack, a spark-free multi-core connector, an explosion-proof button, an explosion-proof detection unit and a three-proof paint circuit board are utilized. Table 2 presents the performance characteristics of the AE instrument.

### 4.3. On-Site Detection of Tank Bottoms

**Figure 6:**
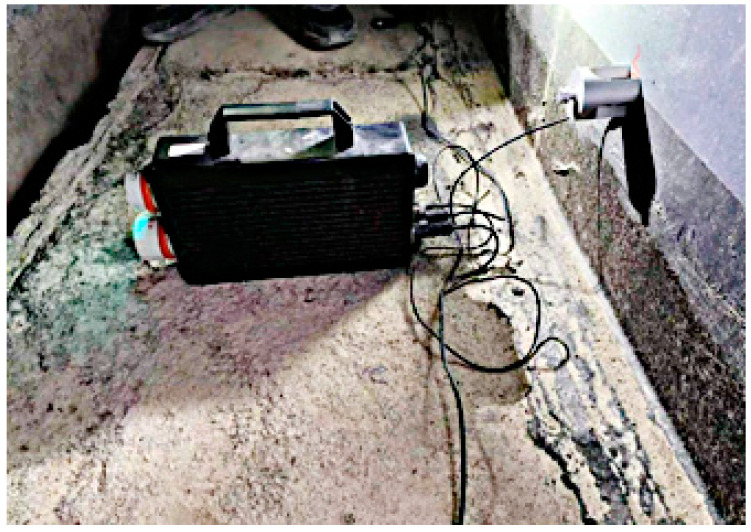
On-site installation of a piezoelectric ceramic sensor.

During this experiment, AE detection was conducted on ten vertical arch oil storage tanks located within an oil depot, and diesel oil constituted the predominant medium stored in these tanks. According to the latest opening and cleaning of the oil tank, the amount of oil sludge in the oil tank was very small compared with that found in a crude oil tank. Considering various factors, 0.2 m was chosen as the installation height. The basic information regarding these tanks is provided below in Table 3:

Before the sensors were installed, surface rust was eliminated from the pre-positioned oil tank wall using sandpaper, and a coupling agent was subsequently evenly applied at the sensor placement location. Finally, a magnetic adsorption fixture was employed to securely attach the sensor to the oil tank wall. The field-work set up is shown in Figure 6.

Before testing, a piezoelectric ceramic sensor was installed on the tank wall for lead break testing, which examines the attenuation characteristics of the oil tank and determines both the maximum spacing between sensors and their arrangement within the tank. Table 4 presents the lead break test results for the B1 tank.

**Figure 7:**
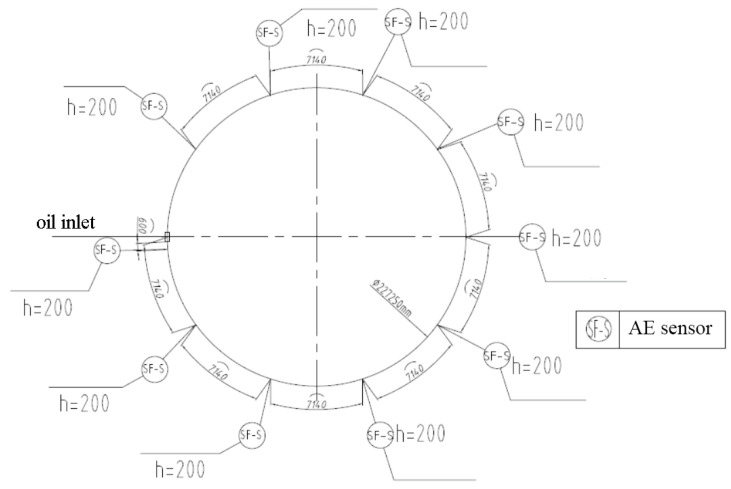
Layout plan of sensors in B-type oil tank.

It can be observed that the maximum effective distance of piezoelectric ceramic sensors amounts to 12 m. Considering factors such as the quantity of AE instruments and the size of the oil tank, the number of sensors, the sensor spacing, and the sensor height in the B-type tanks were set at 10, 7.14 m and 0.2 m, respectively, as shown in Figure 7. Similarly, eight sensors with a spacing of 7.458 m and a height of 0.2 m from the bottom level were used for A-type storage tanks. The D-type storage tanks were equipped with twelve sensors spaced 7.985 m apart, while maintaining a height of 0.3 m from the bottom level.

All of the AE instruments and channels were, respectively, connected to establish synchronization before data acquisition, and the computer that was used for signal processing was moved out of the explosion-proof area. The measured noise level was less than 32 dB, thus setting the acquisition threshold voltage at 35 dB with a gain of 40 dB and a sampling frequency of 1 MHz, respectively.

**Figure 8:**
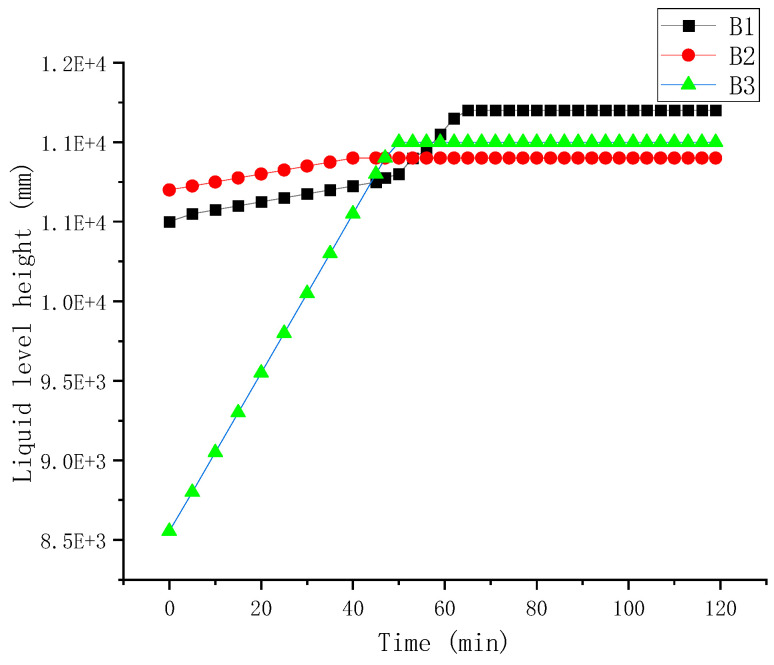
Liquid level height during test.

At the initiation of the experiment, the B1 oil storage tank was first filled with oil up to a height of 11.2 m, which accounted for approximately 97% of its safe capacity level. The entire filling process lasted for 4 h. Subsequently, a two-hour pressure holding test was conducted while AE data was acquired after both the oil pump and valve had been closed. Throughout the test period, there were no changes observed in the liquid level within the oil tank, as depicted in Figure 8.

## 5. Analysis of Experimental Results for AE signals

### 5.1. AE Detection Data of Defects on Tank Floor

After the test, the sensor coordinates were set for AE source localization and detection. Through the AE signals of 10 sensors acquired in the B1 tank, the location information and activity of the AE source could be calculated and obtained, which is the core of carrying out AE detection. Owing to an extensive amount of full-volume signal data, this paper focuses on analyzing selected data from sensor No. 1.

**Figure 9:**
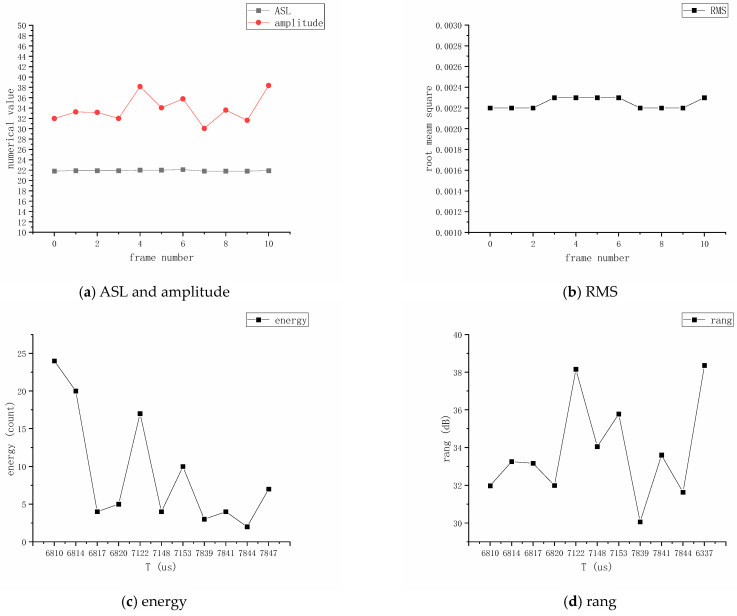
Waveform diagram for AE sensor.

Through analyzing the selected data from sensor No. 1, parameters such as Average Signal Level (ASL) and Root Mean Square (RMS) voltage can be calculated based on occurrence time, amplitude, change patterns, and frequency characteristics. Measuring AE amplitude on a continuous scale makes it exempt from being influenced by thresholds while being suitable for evaluating continuous AE activities. After data processing and the deletion of invalid data based on the energy threshold determined by the lead break test, the resulting waveform is shown in Figure 9. The frame number is the serial number of the acoustic signal collected by the No. 1 sensor, which is sorted according to the timeline to facilitate a comparison of the changes in the energy, ASL and other parameters of different acoustic signals.

The AE signal amplitude fluctuates continuously as time changes; different amplitudes and frequencies occur at different corresponding points. Among them, point 5 is the point with the largest amplitude of 38.16 and the highest number of times, 17. Therefore, it can be determined that the point location can be used as a valid acoustic signal in channel 1. By coupling and analyzing the signal with the data for two or more valid acoustic signals from the other channels in the system, the location information can be judged based on the previous mathematical model.

**Figure 10:**
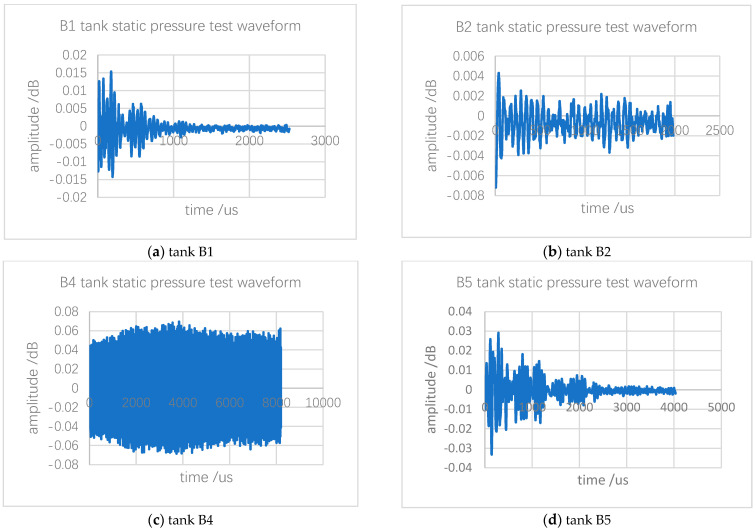
AE static pressure test waveform of storage tank.

The static pressure data for the B1, B2, B4 and B5 tanks are shown in Figure 10.

The data in the figure comes from the data collected by the sensor and were obtained after analysis and processing by the software. These waveforms are mainly used to confirm the signal thresholds of different oil tanks, and obtain the thresholds of the acoustic emission sources of different oil tanks through software analysis, so as to filter out irrelevant signals. The data analysis also reveals the AE signals of tanks under static pressure, and tank background noise levels of all measurement channels was monitored. During the testing of tanks B1, B2, B4 and B5, the noise levels varied slightly to 42.363 dB, 42.123 dB, 42.123 dB and 41.744 dB, respectively. Thus, this can be translated into the detection threshold levels of these tanks.

### 5.2. Analysis of AE Detection Results for Tank Bottoms

**Figure 11:**
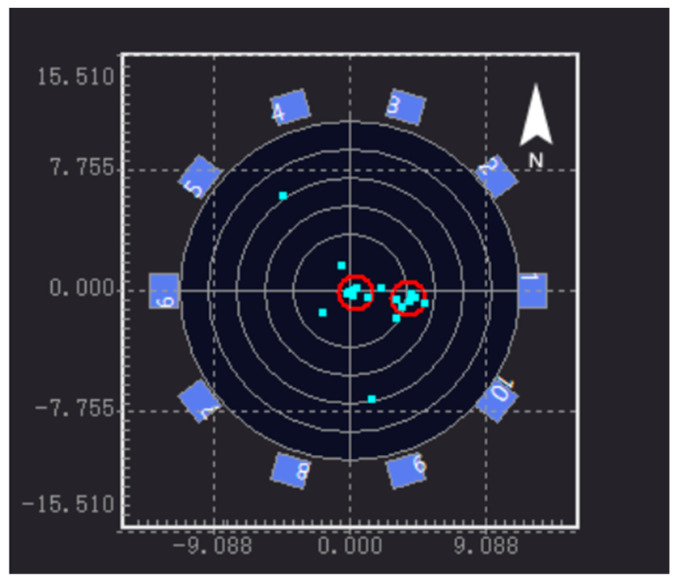
Two-dimensional static pressure AE source localization diagram.

The key preparation for AE detection is determining the acquisition threshold of the sensor; that is, the sensitivity test. The specific method is as follows: In this test, the lead break test was used to test sensitivity. An HB pencil was used to break the lead at a distance of 10 cm from the sensor. If the sensor did not receive a signal, the sensitivity of the sensor was increased until the acquisition threshold was just reached. Every sensor was tested for lead break. According to the obtained acoustic parameters, waveform diagrams of different channels can be obtained. It should be noted that the measurement data should be filtered prior to data analysis to reject noisy data, and the data signals are not related to tank floor corrosion and/or leakage. The plane positioning results of the AE source detection are shown in Figure 11.

**Figure 12:**
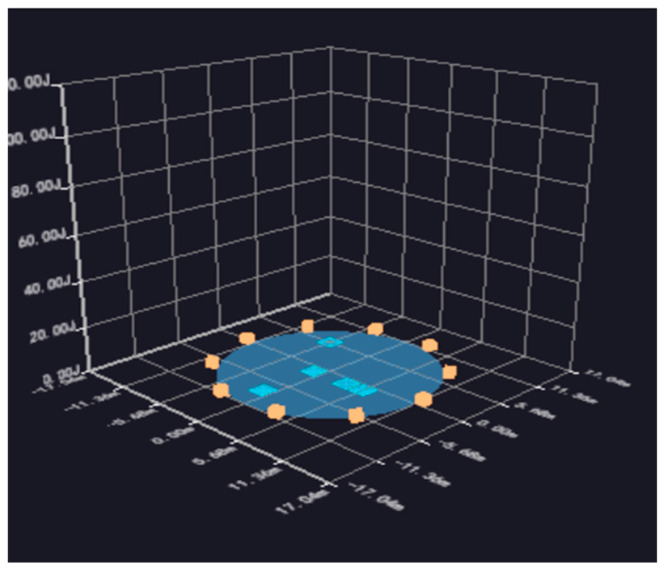
Three-dimensional static pressure AE source localization diagram.

1–10 represents the number of sensors, and the blue and red circles represent the location of the sensor and the concentration area of the acoustic emission source, respectively. One can see that the AE sources are mainly distributed in the center and on the right side of the oil tank, showing a spot-like and concentrated distribution. It can be judged that there may be a relatively serious amount of flaky corrosion in this area, which is the key location for opening inspection or maintenance of the oil tank. In addition, there is an AE source signal in the upper left and lower right, respectively, indicating that there may be spot-like corrosion in this area; alternatively, it may be an invalid signal, which needs to be confirmed by other detection methods such as magnetic leakage and ultrasound after opening the tank. Based on the analysis of the sensor data, information about the location of the acoustic emission signal sources is obtained and assessed according to their distribution. The blue area in Figure 12 is the area in which the acoustic emission signal sources are gathered in the 3D model, and its height represents the number of acoustic signal sources.

According to the AE source’s coordinates and quantity information imported from the figure, it is concluded that the center (0, 0) coordinates on the tank’s bottom are the origin in area a. There were six effective AE sources in the coordinates (X: 3.95 m, Y: −0.50 m, radius: 1.14 m) of area A, and seven effective AE sources in area B (X: 0.49 m, Y: −0.23 m, radius: 1.14 m). The time difference in the positions of the AE sources on the tank’s bottom plate were analyzed and graded.

The tank’s bottom plate is divided into different evaluation areas with a length that is no greater than 10% of the diameter, and the time difference between the locations of the events obtained in the evaluation area have been analyzed and calculated. According to Table 5, C< E ≤ 10C (C is five in this time, obtained through the preliminary test), the time difference in the location of the AE source of the tank bottom plate has been analyzed and graded, and it is concluded that the B1 storage tank is grade II. The tank is mildly corroded.

Similarly, the AE test was performed on the remaining nine oil tank bottom plates, according to the above steps, and the test results are shown in Table 5.

### 5.3. Open Tank Detection

**Figure 13:**
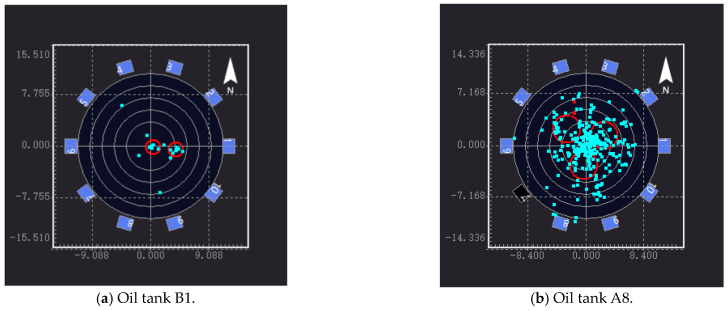

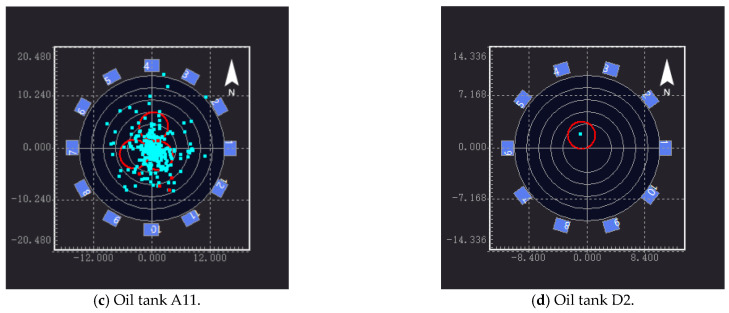
AE detection results for the 4 oil tanks.

**Figure 14:**
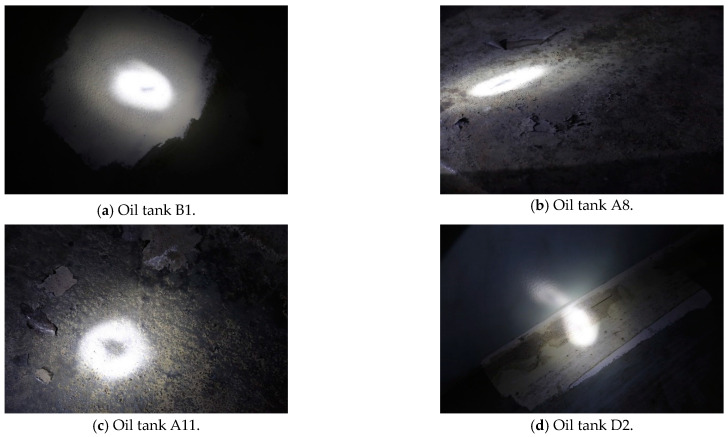
Open detection results for the 4 oil tanks.

Since the 10 oil tanks are all used as petrochemical equipment, the testing process needs to be determined according to the actual storage state and working requirements. In this study, only parts of the oil tanks were tested by opening the tanks. The B1, A8, A11, and D2 oil tanks were selected to be opened for detection. Figure 13 depicts the AE detection results of the four oil tanks. Figure 14 depicts open detection results of the four oil tanks. By comparing the two methods, the acoustic emission efficiency of the tank bottom plates can be obtained effectively.

The upper surfaces of the bottom plates of the four oil tanks were tested by opening the tanks. It can be seen from the picture that no relevant corrosion was found on the upper surface of the bottom plate of the B1 oil tank, and the upper surface was smooth and flat. Corrosion points were found on the upper surface of the A8 tank’s bottom, and no serious corrosion conditions such as corrosion perforation or weld oil leakage were found. Corrosion points were found on the bottom of the A11 oil tank. There were pits, peeling paint, floating rust, etc. The corrosion position was a little to the right of the center, and there was a corrosion pit in the upper part of the center. The paint on the upper right side of the center was partially cracked and peeled, and was is floating rust; no associated corrosion was found on the upper surface of the D2 tank’s floor.

Due to the limited experimental conditions, it was difficult to carry out excavation detection on the lower surface. Combined with the AE source location function, the detection conditions of open tanks were compared, and it was found that the prediction of the corrosion area of the tank floor was very consistent with the actual corrosion situation, and the regional positioning was also practically consistent, indicating that the AE instrument could effectively predict the corrosion situation in the oil tank. The AE test results for the oil tanks were compared with the results of the opening tests, as shown in Table 6.

It can be seen that the AE instrument testing of tank B1 evaluated it as more severe, while the detection results of the other three oil tanks are consistent. Overall, the open tank test results suggest that the AE instrument’s detection and evaluation results are conservative. The AE instrument’s positioning of the corrosion area of the tank bottom plate is principally the same as that of the practical corrosion area, indicating that the AE instrument shows good positioning performance for the positioning of the corrosion area in the oil tank. In conclusion, the detection and evaluation of the AE instrument can ensure the safe operation of the oil tank, and can be used as the main basis for the regular maintenance of an oil tank.

## 6. Conclusions

The weakest and most dangerous area of an oil tank is the floor, and the most common causes of failure are corrosion and welding defects. In this paper, an explosion-proof AE instrument was designed for corrosion detection on a storage tank’s bottom plate. Ten oil tanks that are currently in use were tested, and four of them were tested using open-tank tests. The main conclusions are as follows.

(1) The AE detection can reflect the corrosion on the oil tank’s floor accurately. The instrument locates the AE source, which greatly reduces the workload compared to traditional can-opening detection. According to the comparative analysis of the inspection results before and after opening, the detection operation can be carried out under the condition that the vertical steel oil tank has oil inside it. The detection data provided are basically consistent with the results of the opening detection tests and have a strong guiding effect on determining the opening order of oil tank maintenance.
(2) This study designed an AE instrument for use in cave oil tanks with high explosion-proof requirements. The electronic zero devices used in the instrument meet the oil and gas explosion-proof requirements, and the parts and equipment meet the national explosion-proof standards. AE detection can be carried out on the oil tank under the premise of explosion-proof safety.
(3) The experiments show that tank floor detection is a complex and dangerous task. The AE technology analyzes the emission source classification through time difference positioning and regional positioning. AE detection classifies the corrosion status of the tank floor according to the number of events per unit time per unit area and the number of impacts per unit of time, thereby qualitatively reflecting the corrosion status of the tank floor. The event number index can accurately describe the corrosion status of the bottom plate.

## Figures and Tables

**Table 1:** Classification of AE sources based on time difference location analysis.

The Source Level	The Number of Positioning Events E per Hour in the Evaluation Zone	Evaluation of the Evaluation Area’s Corrosion State
I	E ≤ C	No signs of local corrosion
II	C < E ≤ 10C	Slight signs of localized corrosion
III	10C < E ≤ 100C	Obvious signs of localized corrosion
IV	100C < E ≤ 1000C	Minor indications of severe localized corrosion
V	E > 1000C	Signs of severe localized corrosion

**Table 2:** Performance parameters of explosion-proof AE instrument.

Type	Parameter
Sampling rate	≥1 MHz
Number field of channels	1 × 15
Test frequency range	30~60 kHz
Storage capacity	≥32 GB
Communication interface	Network interface/100 M
Time of continuous work	≥6 h
Explosion-proof identification	All certified to meet
Explosion-proof battery	FRT-FB183S01C
Explosion-proof AE sensor	AE503S (Ex ibIIA T3 Gb)
Explosion-proof non-spark type multi-core connector	16YT-30J/GZ-30K (Ex nAIIC T4 Gc)
Explosion-proof button	YH8030 (Ex deIIC Gb)
Explosion-proof AE detection unit	ExAEM
Explosion-proof distribution device	BX1-23 (Ex deIIC T4)

**Table 3:** Basic information about the oil tanks.

Number	Volume (m^3^)	Medium	Sediment Information	Working Temperature (°C)	Safe Altitude (m)	Diameter (m)
B1	5000	diesel oil	few	3	11.5	22.725
B2	5000	diesel oil	few	10	11.5	22.725
B4	5000	diesel oil	few	11	11.5	22.725
B5	5000	diesel oil	few	11	11.5	22.725
B7	5000	diesel oil	few	10	11.5	22.725
A8	3000	aviation kerosene	few	8	9.5	19.06
A11	3000	diesel oil	few	8	9.5	19.06
D2	10,000	diesel oil	few	9	12.8	30.5
D3	10,000	diesel oil	few	10	12.8	30.5
D3	10,000	diesel oil	few	11	12.8	30.5

**Table 4:** B1 oil tank lead break test data.

(a) Basic parameters of lead break test									
background noise/dB				<32					
threshold level/dB				35					
gain/dB				40					
Maximum sensor spacing/m				12					
Attenuation measurement sensor number				No. 1					
(b) Signal attenuation test									
Simulated source distance/m	0.5	1	1.5	2.0	3.0	4.0	6.0	9.0	12.0
signal amplitude/dB	61	57	54	51	44	42	40	38	37

**Table 5:** Results of AE tests on the bottom plates of the oil tanks.

Number	Classification	Evaluation
B1	II	slight signs of localized corrosion
B2	II	slight signs of localized corrosion
B4	II	slight signs of localized corrosion
B5	II	slight signs of localized corrosion
B7	II	slight signs of localized corrosion
A8	II	slight signs of localized corrosion
A11	II	slight signs of localized corrosion
D2	I	No signs of local corrosion
D3	I	No signs of local corrosion
D4	I	No signs of local corrosion

**Table 6:** Comparison between AE test results and open tank test results of oil tanks.

Number	Medium	AE Test Results	Open Tank Test Results	Comparison of Corrosion Location
B1	diesel oil	II	No obvious corrosion	The corrosion location is different
A8	aviation kerosene	II	Slight corrosion	The corrosion location is same
A11	diesel oil	II	Slight corrosion	The corrosion location is same
D2	diesel oil	I	No obvious corrosion	The corrosion location is same

## Data Availability

Data are contained within the article.
